# The Prevalence of Positive Fungal Cultures in Patients with Chronic Rhinosinusitis in a High Altitude Region of Iran 

**Published:** 2012

**Authors:** Farnaz Hashemian, Farshad Hashemian, Mohammadhossein Bakhshaei

**Affiliations:** 1 *Assistant professor of Hamadan University of Medical science ,otolaryngology department *; 2 *Islamic Azad University, Pharmaceutical Sciences Branch,Clinical Pharmacy Department *; 3 *Assistant professor of Hamadan University of Medical science ,Anesthesiology department*

**Keywords:** Chronic rhinosinusitis, Fungus, Altitude region

## Abstract

**Introduction:**

There are suspended fungal spores in the air and in the nasal mucosa of adults, especially in areas with a humid climate. Several studies have revealed the role of fungi in the pathogenesis of chronic rhinosinusitis (CRS) in recent years but it is a topic of controversy, especially in regions with low humidity. The aim of this study was to evaluate the prevalence of fungal species in intraoperative specimens from patients who underwent functional endoscopic sinus surgery (FESS) for CRS in Hamadan, a high altitude region of Iran.

**Materials and Methods:**

In this prospective cross-sectional study specimens were obtained from 62 patients with a diagnosis of CRS according to clinical and computed tomography criteria who underwent endoscopic sinus surgery. During the functional endoscopic sinus surgery, specimens were collected from the nose and sinuses of patients and preserved in conical centrifuge tubes containing Sputolysin and chloramphenicol. The specimens were then plated on Sabouraud dextrose agar, Mycosel agar, Niger seed agar, and Chrom Agar/Candida plates and incubated at 30°C for up to 1 month. At the end of the incubation period the samples were evaluated microscopically to detect fungi and identify their genera and species.

**Results:**

The fungal cultures were positive in 16 out of 62 patients with CRS (25.8%). In order of frequency the fungal genera and species were: Aspergillus fumigatus (9), Aspergillus niger (3), Candida albicans (2), Penicillium sp. (1) and Cladosporium sp. (1). The percentage of positive cultures collected was higher in winter but the difference was not statistically significant compared to the rest of the year.

**Conclusion:**

Our data show that 25.8% of patients tested positive for the presence of fungi. The results strengthen the theory regarding the role of fungi in the pathogenesis of CRS even in areas with low humidity. Aspergillus was the most commonly isolated fungus.

## Introduction

Chronic rhinosinusitis (CRS) is defined as an inflammatory disease of the nasal and paranasal mucosa where symptoms persist for longer than 3 months. The ultimate end-stage of the chronic inflammation is polypoid mucosal thickening and nasal polyps. Chronic rhinosinusitis is the most common chronic disease in many countries (1). According to one report, 14.1% of the population of the USA has CRS, making it more common than arthritis (12.5%), orthopedic impairment (12.1%), and hypertension (11.4%) (2). 

Fungi are found mainly in air, dust, soil, plants, and decaying organic matter. They adhere to dust particles and are inhaled and deposited on the nasal and paranasal sinus mucosa. The warm, moist environment of the upper respiratory tract is an ideal environment for the proliferation of these organisms (3). Review of the current literature appears to offer strong evidence to support both allergic and nonallergic forms of noninvasive fungal inflammation (4). Ponikau and colleagues suggest that the immunological response to the presence of fungi in the sinonasal cavity has a role in the pathogenesis of CRS. They were able to detect fungi in 93% of patients with CRS who underwent endoscopic sinus surgery by using an exquisitely sensitive culture technique (5). Ponikau and Tichenor hypothesized that these patients with CRS had activated eosinophiles, which release a product called major basic protein (MBP) into the mucus that attacks and kills the fungus but is very irritating to the lining of the sinuses. The consequence of the release of MBP is damage to the lining of the sinuses that then allows bacteria to proliferate (6). 

Although fungi are ubiquitous, the degree of fungal exposure appears to vary based upon environmental conditions. Moisture and temperature appear to be the most important determinants affecting potential fungal growth (7). The aim of this study was to evaluate the prevalence of fungi in intraoperative specimens from patients who underwent functional endoscopic sinus surgery for CRS in a high altitude region of Iran where there is a low level of humidity. 

## Materials and Methods

This prospective cross-sectional study was performed on specimens obtained from 62 patients with CRS, who underwent endoscopic sinus surgery at Besat Hospital, Hamadan, Iran. CRS was diagnosed based on the American Academy of Otolaryngology-Head and Neck Surgery (AAO-HNS) definition of CRS (7). Demographic data for the patients are shown in (Table 1). 

Immunosuppressed patients and those with invasive fungal sinusitis were excluded from the study. No patients had received topical or systemic corticosteroids for at least four weeks prior to sample collection. All patients filled out a consent form to participate in the research and the study was approved by the ethics committee of Hamadan University of Medical Sciences.

**Table 1:** Distribution of patients with CRS who underwent FESS according to age and gender

**Age**	**Gender**		**(%) ** **n** **Total**
	**(%) ** **n** **Female**	**(%) ** **n** **Male**	
10–19	4(6.5)	5(8.1)	9(14.5)
20–29	6(9.7)	8(12.9)	14(22.6)
30–39	7(11.3)	11(17.8)	18(29.1)
40–49	3(4.9)	8(12.9)	11(17.8)
50–59	1(1.6)	4(6.5)	5(8.1)
≥60	2(3.2)	3(4.9)	5(8.1)
Total	23(37.1)	39(62.9)	62(100)

Specimens from the patients’ nose and sinuses, including mucin, polypoid material, and sinus mucosa, were collected intraoperatively and tissue samples were cut into small pieces using sterile scissors. The samples were preserved in conical centrifuge tubes containing Sputolysin (diluted in N-acetyl-l-cysteine) and chloramphenicol (0.5 µg/mL) and then centrifuged for 10 min. The supernatant was discarded and the sediments plated on Sabouraud dextrose agar, Mycosel agar, Niger seed agar, and Chrom agar/Candida plates and incubated at 30°C for up to 1 month. At the end of the incubation period the plates were evaluated microscopically in order to detect the fungi and identify their genera. Demographic data, such as the age and sex of the patients and the season when the specimens were collected, were recorded and analyzed using SPSS software.

## Results

The fungal cultures were positive in 16 of the 62 patients with CRS (25.8%). The specimens were obtained from the mucosa of the paranasal sinuses in 38 cases (61.3%), from polyps in 19 cases (30.7%), and from intracavitary secretions in 5 cases (8.1%) (Table 2).

**Table 2:** Sample types taken from patients with CRS who underwent FESS

Type of samples	**(%)n **
Mucosa	38(61.3)
Polyp	19(30.7)
Secretion	5(8.1)
Total	62(100)

Sampling locations included the maxillary sinuses and middle meatus in 32 cases (50.1%), the ethmoid sinuses in 22 cases (35.4%), the sphenoid sinuses in 5 cases (8.1%), and the frontal sinuses in 3 cases (6.4%) (Table 3). 

**Table 3:** Location of samples taken from patients with CRS who underwent FESS

**Location of sample**	**n(%)**
Maxillary sinus & middle meatus	32(50.1)
Ethmoid sinus	22(36.4)
Sphenoid sinus	5(8.1)
Frontal sinus	3(6.4)
Total	62(100)

The genus and species of the fungi in order of frequency were: Aspergillus fumigatus (9), Aspergillus niger (3), Candida albicans (2), Penicillium sp. (1), and Cladosporium sp. (1) (Table 4). 

**Table 4:** Fungi isolated from patients with CRS who underwent FESS

**Fungus isolated**	**n (%)**
Aspergillus fumigatus	9(56.3)
Aspergillus niger	3(18.8)
Candida albicans	2(12.5)
Penicillium sp.	1(6.3)
Cladosporium sp.	1(6.3)
Total	16(100)

The percentage of positive cultures collected was higher in winter but this difference was not statistically significant compared to the other seasons (P=0.091) (Table 5).

**Table 5:** Incidence of positive fungal culture according to the season of sample collection

Season	No. of patients with CRS who underwent surgery	No. of positive cultures	Percentage of patients infected
Spring	9	2	22.2%
Summer	15	4	26.7%
Fall	23	5	21.7%
Winter	15	5	33.3%

P =0.091

## Discussion

In the past decades fungal infections in humans have seemed to increase. Various factors may play a role in this trend, such as the increased use of corticosteroids and immunosuppressant drugs in cancers and transplantations. In addition, diabetes mellitus, the use of broad spectrum antibiotics, the human immunodeficiency virus (HIV), and other unknown etiologies may also be associated with the increasing incidence of fungal infections. A study performed at the Mayo Clinic hypothesized a stronger role for fungi in the pathogenesis of chronic rhinosinusitis (5) but while much published data supports this hypothesis, other studies reject it and, until now, the controversies have continued. 

According to a study by Shin and colleagues, positive cultures for fungi were obtained in 60 out of 108 patients with CRS (63.0%), and 28 out of 45 normal volunteers (62.2%) (8). that Also, in Ireland Hafidha and colleagues showed fungi can be cultured from the nasal mucous of healthy control subjects (70%) as well as from patients with CRS (47%) (9). Lebowitz and colleagues were able to demonstrate the presence of fungi in 56% of intraoperative specimens obtained from 55 patients undergoing surgery for CRS (10). On the other hand, Dall’Igna and colleagues in Brazil isolated fungus in 6.7% of patients (11), Nigro and colleagues found fungus in only 2.4% of patients (12), and Granville and colleagues found fungus in 12% of b

Similarly, Foreman and colleagues showed that 11 out of 50 CRS patients (22.0%) had characteristic fungal biofilms (14). In Iran, Hedayati and colleagues showed that fungus could be cultured from 70.0% of patients with CRS undergoing endoscopic sinus surgery in Sari, a city with high humidity in the north of Iran. In that study Aspergillus was the most commonly isolated fungus (45.0%) (15).

In the present study, which was performed in Hamadan, one of the highest altitude cities in Iran where there is a low level of humidity, fungal cultures were positive in 16 out of 62 patients with CRS (25.8%) and Aspergillus species (Niger and Fumigatus) were isolated in 75% of the positive cultures. Luong and Marpel showed an increased prevalence of respiratory symptoms among children and adults living in humid conditions but the role of humidity as the sole factor in fungal exposure is not clear. However, it seems that environmental factors such as moisture and temperature can at least be a factor in the presence of fungi (10). According to a study by Shin and colleagues, fungus culture rates were higher during summer and fall and Cladosporium, Aspergillus, Alternaria, and Penicillium were frequently isolated from CRS patients and normal volunteers (8). The percentage of positive cultures in the present study was higher in winter but this was not a statistically significant result (P=0.091). However, in agreement with Shin and colleagues Aspergillus fumigatus, Aspergillus niger, Candida albicans, Penicillium sp., an Cladosporium sp. were isolated from the samples. 

In another study conducted on 639 patients by Dennis it was shown that CRS and systemic fungal symptoms are likely caused by an immune response to fungal antigens. After removal of fungal antigens from the nose and the air the immune reaction stopped and the sinus mucosal edema, polyps, pus, and systemic symptoms improved or resolved. The study suggested that fungus is probably responsible for most CRS and for a variety of systemic symptoms as a result of a genetic defect in the T cell receptor Vbsite (16).

## Conclusion

It seems environmental and geographical conditions are factors in fungus-induced CRS. Also methods of culture may be important in the detection of fungi. In the present study, fungal cultures were positive in 25.8% of patients with CRS. The majority of fungi cultured were of the genus Aspergillus. As Hamadan is a high altitude region, located 1800 m above sea level with a low level of humidity, the amount of fungal infection is a noticeable 
